# Does radiation therapy increase gadolinium accumulation in the brain?: Quantitative analysis of T1 shortening using R1 relaxometry in glioblastoma multiforme patients

**DOI:** 10.1371/journal.pone.0192838

**Published:** 2018-02-14

**Authors:** Woo Hyeon Lim, Seung Hong Choi, Roh-Eul Yoo, Koung Mi Kang, Tae Jin Yun, Ji-Hoon Kim, Chul-Ho Sohn

**Affiliations:** 1 Department of Radiology, Seoul National University College of Medicine, Seoul, Korea; 2 Center for Nanoparticle Research, Institute for Basic Science (IBS), Seoul National University, Seoul, Korea; 3 School of Chemical and Biological Engineering, Seoul National University, Seoul, Korea; Technische Universitat Munchen, GERMANY

## Abstract

**Objective:**

This study evaluated the possibility of accelerated gadolinium accumulation in irradiated brain parenchyma where the blood-brain barrier was weakened.

**Methods:**

From January 2010 to June 2015, 44 patients with supratentorial glioblastoma were retrospectively identified who underwent pre- and post-radiation brain MR imaging, including R1 mapping. The mean dose of administered gadobutrol (Gadovist, Bayer, Germany) was 5.1 vials. Regions of interest (ROIs) were drawn around tumors that were located within 50–100% iso-dose lines of maximum radiation dose. ROIs were also drawn at globus pallidus, thalamus, and cerebral white matter. Averages of R1 values (unit: s^-1^) before and after radiation and those of R1 ratio (post-radiation R1 / pre-radiation R1) were compared by t-test or rank sum test as appropriate. Multiple linear regression analysis was performed to evaluate independent association factors for R1 value increase at irradiated parenchyma.

**Results:**

The mean R1 values in peri-tumoral areas were significantly increased after radiotherapy (0.7901±0.0977 [mean±SD] vs. 0.8146±0.1064; P <.01). The mean R1 ratio of high radiation dose areas was significantly higher than that of low dose areas (1.0055±0.0654 vs. 0.9882±0.0642; P <.01). The mean R1 ratio was lower in those who underwent hypofractionated radiotherapy (mean dose, 45.0 Gy) than those who underwent routine radiotherapy (mean dose, 61.1 Gy) (0.9913±0.0740 vs. 1.0463±0.0633; P = .08). Multiple linear regression analysis revealed that only radiotherapy type was significantly associated with increased R1 (P = .02) around tumors.

**Conclusions:**

Radiotherapy can induce R1 value increase in the brain parenchyma, which might suggest accelerated gadolinium accumulation due to damage to the blood-brain barrier.

## Introduction

Gadolinium (Gd)-based contrast agents (GBCAs) are widely used contrast materials for magnetic resonance (MR) imaging in clinical setting. Recently, GBCAs are attracting attention to many radiologists after Kanda et al [1] and Errante et al [2] reported hyperintensity in the dentate nucleus (DN) and globus pallidus (GP) on unenhanced T1-weighted image (T1WI) might be related with usage of GBCAs. Subsequent studies [3–8] revealed that hyperintensity in the DN on T1WI was associated with previous administration of linear GBCAs, whereas previous administration of macrocyclic GBCAs showed no such association.

These studies [1–7] suggested that the mechanism of Gd deposition in the brain is the dissociation of the Gd from its chelate molecule. However, no study has examined why the DN and GP consistently show remarkable T1 hyperintensity, while other parts of the brain show less significant T1 hyperintensity.

Meanwhile, the blood-brain barrier (BBB) functions as a barrier between blood and brain parenchyma that controls brain microenvironment by regulating transmembranous transportation [9]. Several conditions including irradiation [10–16] could result in disruption of BBB.

We hypothesized that the integrity of the BBB might contribute to the selective deposition in the brain. No study has investigated the relationship between BBB damage and Gd deposition in the brain. Thus, the purpose of this study was to evaluate the absolute R1 (unit: s^-1^) changes after administration of macrocyclic GBCA in glioblastoma (GBM) patients who underwent radiotherapy, which can induce BBB weakness.

## Materials and methods

Institutional Review Board at Seoul National University Hospital approved this study. The requirement of written informed consent was waived because this was a retrospective study.

### Patients

From January 2010 to June 2015, a group of 2148 consecutive patients who underwent brain MR imaging under suspicion of brain tumor (protocol name: Glioma study) at our institution were identified. All brain MR images included contrast-enhanced T1WI using macrocyclic GBCA (gadobutrol, Gadovist, Bayer, Leverkusen, Germany).

Among the patients, we enrolled those who met the following inclusion criteria: (a) patients who underwent gross total removal of tumor, defined as absence of measurable enhancing lesion on immediate post-operative MR imaging [17], in our institute to confirm histopathological diagnosis of GBM; (b) patients who completed concurrent chemoradiotherapy with temozolomide followed by adjuvant temozolomide; and (c) patients who underwent pre- and post-radiation brain MR imaging including R1 map source images. Of the 2148 patients, 107 patients satisfied inclusion criteria (a) and (b), and 50 patients met all three inclusion criteria.

**Fig 1:**
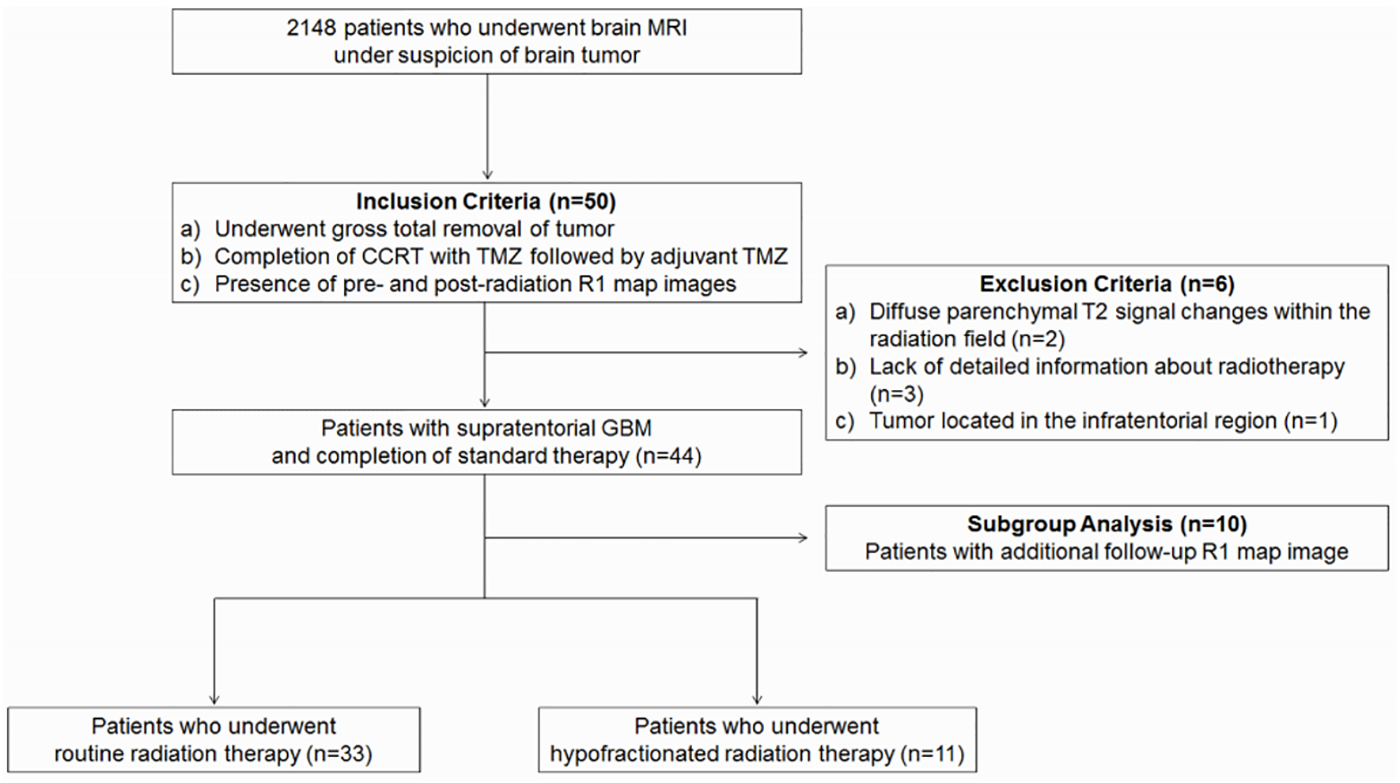
Flow chart of the study population. Note: MRI = magnetic resonance imaging, CCRT = concurrent chemoradiotherapy, TMZ = temozolomide, GBM = glioblastoma multiforme.

The data from 6 patients were excluded from analysis for the following reasons: (a) two patients had diffuse parenchymal T2 signal changes within the radiation field; (b) three patients did not have detailed information about radiotherapy because they underwent radiotherapy in other hospitals; and (c) one patient had a tumor located in the infratentorial region, where the aliasing artifacts were frequently observed. Finally, 44 patients with supratentorial GBM were enrolled in our study. Fig 1 summarizes the flow chart for enrollment of the study population. Disease progression after the initial chemoradiotherapy was determined according to RANO criteria [17].

### MR imaging protocol

**Fig 2:**
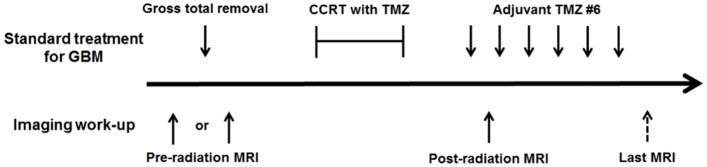
Timeline of patient treatment and the imaging study. The standard treatment for newly diagnosed GBM includes gross total removal of the tumor and concurrent chemoradiotherapy with temozolomide, followed by adjuvant temozolomide. In this study, we enrolled patients who underwent pre- and post-radiation brain MRI including R1 map source images. Pre-radiation MRI included MRI performed either before or after tumor removal. The mean time interval between pre- and post-radiation MRI session was 4.2 ± 2.1 months. Further follow-up brain MRI with R1 mapping was available for 10 patients.

The timeline of patient treatment and imaging studies is described in Fig 2. MR imaging was performed with a 3.0 T imaging unit (Verio or Skyra, Siemens, Erlangen, Germany) with a 32-channel head coil.

For axial R1 map acquisition, we used a volumetric interpolated breath-hold examination (VIBE) sequence (repetition time msec/echo time msec, 4/1.4; section thickness, 3 mm; field of view, 240 x 240 mm; matrix 192 x 192; number of excitations, 1; echo train length, 1) using 3 different flip angles: 2, 8, and 15 degrees.

The imaging sequences of the brain also included pre- and post-contrast T1WI using magnetization-prepared 180 degree radio-frequency pulses and rapid gradient-echo (MP-RAGE)(1420 to 1500/1.9; 1 mm; 223 x 223 mm to 249 x 249 mm; 240 x 240 to 256 x 256; 1; 1), fast spin-echo T2-weighted images (5160/91 to 6440/114; 5 mm with 1 mm gap; 175 x 220 mm to 199 x 220 mm; 640 x 255 to 640 x 290; 3; 19), and fluid-attenuated inversion-recovery (9000/97; 5 mm with 1 mm gap; 175 x 220 mm to 199 x 220mm; 384 x 184 to 384 x 209; 1; 15) images. Post-contrast T1WI was performed after the intravenous administration of gadobutrol at a dose of 0.2mmol/kg of body weight.

### R1 map generation

Using the variable flip angle technique with spoiled gradient echo sequences, the T1 value of tissue was calculated by the following equation:

$$SI\;=\;S_{0}∙\frac{\text{sin}\;\alpha ∙(1-S_{1})∙S_{2}}{1-S_{1}∙\text{cos}\;\alpha }$$

where *S*_1_ = exp(−TR/T1) and S_2_ = exp(−TE/T2*) (SI = signal intensity; S_0_ = the equilibrium magnetization; T1 = longitudinal relaxation; T2* = effective transverse relaxation; TR = repetition time; TE = echo time; α = flip angle). If TE is much shorter than T2*, S_2_ will converge to 1 [18], and the equation can be simplified to the following linear form:

$$\frac{SI}{\text{sin}\;\alpha }\;=\;S_{1}∙\frac{SI}{\text{tan}\;\alpha }+S_{0}∙(1-S_{1})$$

We could calculate the T1 value of the tissue from the slope (S_1_) of this equation [18–20]. The R1 value of the tissue could thus be measured as the reciprocals of the T1 value.

The source images of the R1 maps using three different flip angles were transferred from the picture archiving and communication system (PACS) workstation to a software package for image analysis (Nordic ICE, Nordic Neuro Lab, Bergen, Norway). The source images of the R1 maps were stored in NIfTI-1 (nii) format. Using three NIfTI-1 files for each patient, the R1 map was generated to perform quantitative analysis.

### Data analysis

**Fig 3:**
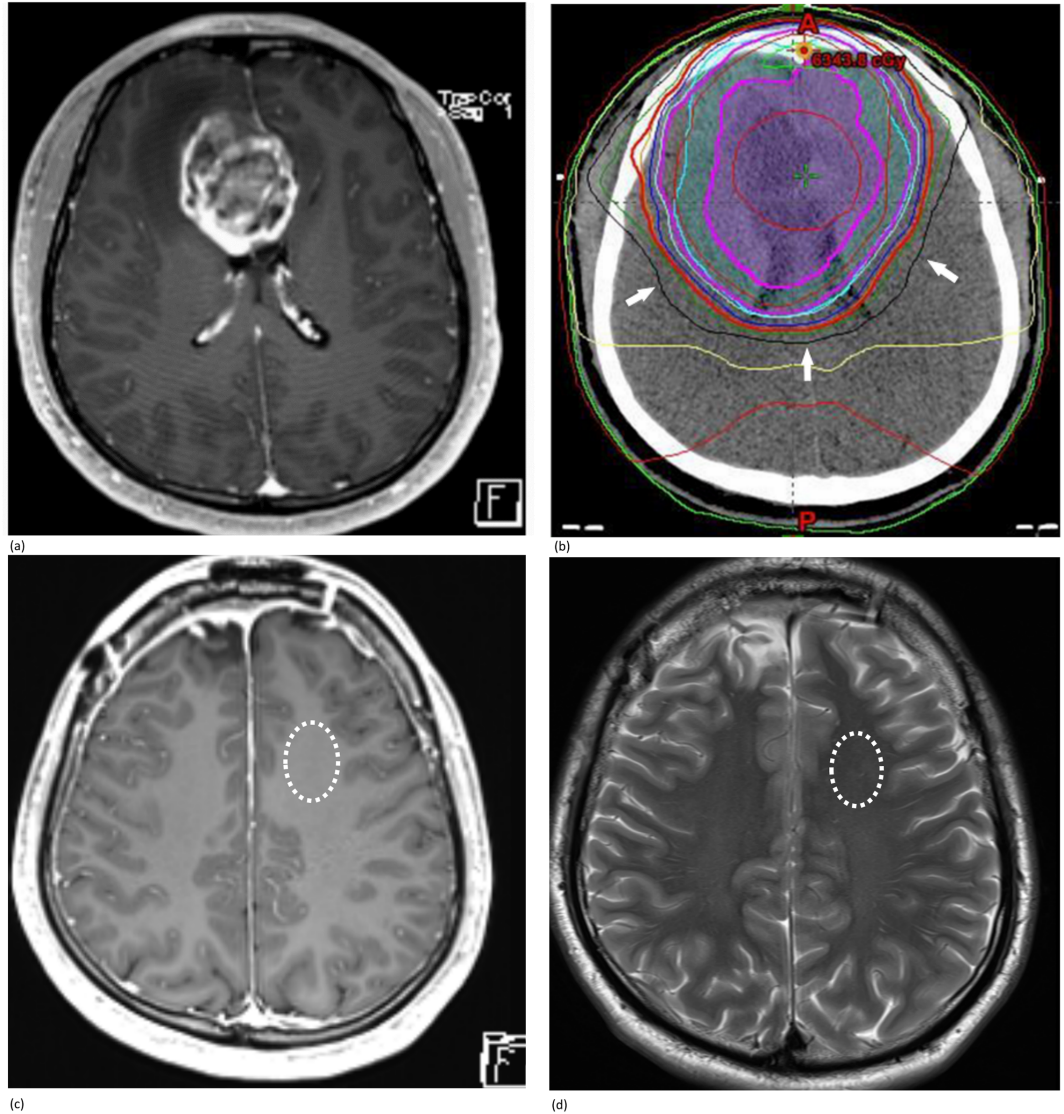
ROI around primary tumors. A 28-year-old male patient with severe headache for 1 month underwent brain MR imaging. **(a)** Contrast-enhanced axial T1WI revealed a heterogeneous enhancing mass located at the right frontal lobe. He underwent gross total removal of the brain tumor, and final pathology confirmed GBM. He also completed radiotherapy (total dose = 61.2 Gy). **(b)** The black line (arrows) in the radiotherapy plan map showed the iso-dose line of 3600 cGy, which was the 56.7% iso-dose line of the maximum dose (6343.8 cGy). For R1 measurement, the ROI was drawn at the peri-tumoral area located within the 50–100% iso-dose line of the maximum dose and both **(c)** overt enhancing area and **(d)** abnormal T2 hypersignal intensity area were avoided.

S1 Fig summarizes the diagram of the study design. All the R1 measurements were performed independently by two different observers (W.H.L, S.H.C; 2 and 15 years of experience in neuroradiology, respectively). For the R1 measurement in the peri-tumoral area, the mean value of three regions of interest (ROIs; 93.2±37.8 mm^2^ [mean±SD]) was used. The ROIs were drawn around the primary tumors within 50–100% iso-dose lines of the maximum radiation dose. Both overt enhancing area and abnormal T2 signal intense area were avoided for R1 measurements (Fig 3). The distances between the three ROIs were at least 2 cm. The ROIs were also drawn at the GP and thalamus with maximal coverage of those areas on the axial scan. Frontal, parietal, and temporal white matter were also included in the R1 measurements, for which the ROIs were 143.2±69.4 mm^2^. In white matter with high T2 signal intensity, the ROIs were not drawn, and the area was considered unmeasurable. The DN was excluded from R1 value measurement because R1 values were significantly incorrect due to aliasing artifact. The ROIs were drawn only once for each brain area other than the peri-tumoral region.

**Fig 4:**
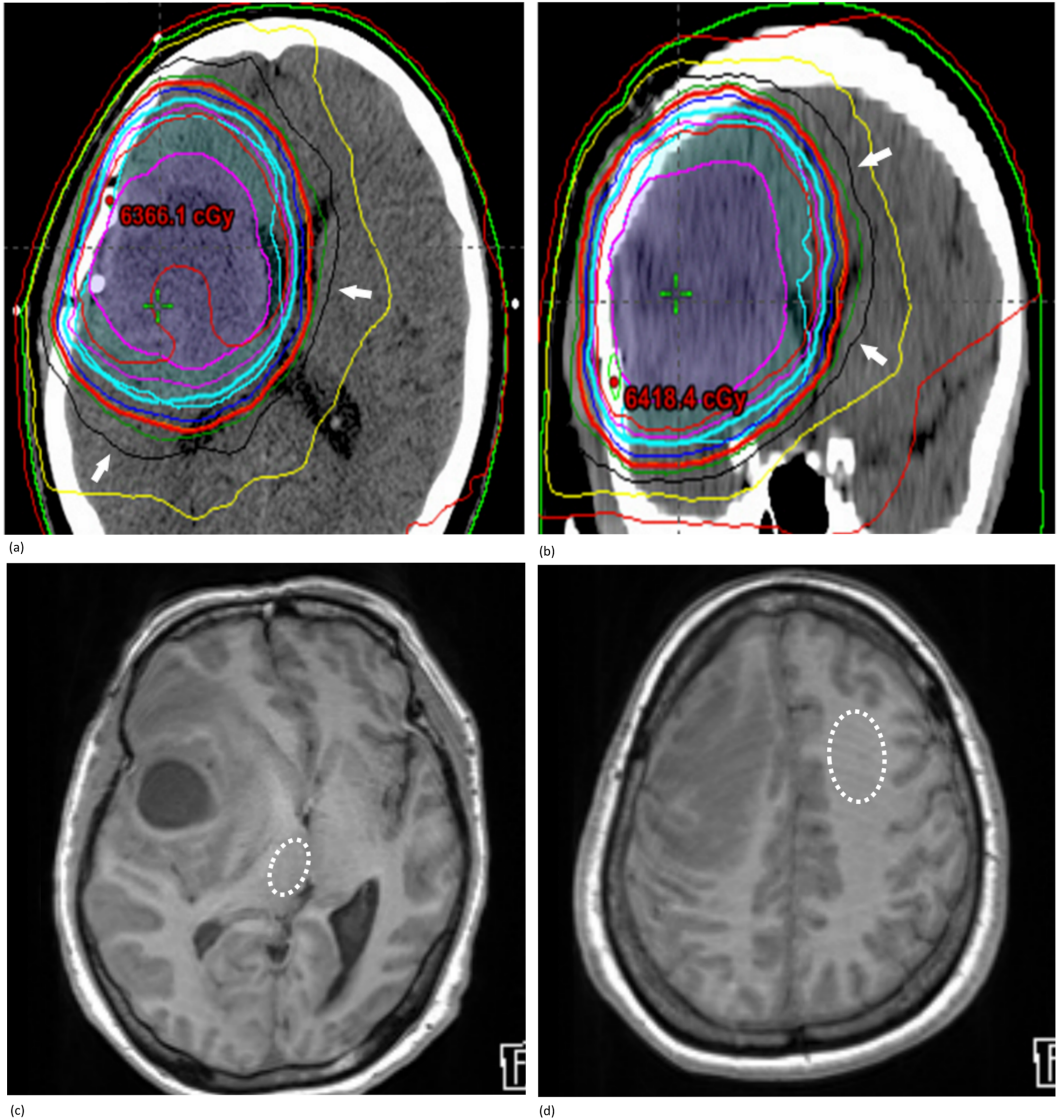
Definition of Area_H_ and Area_L_. A 58-year-old female patient underwent radiotherapy for the treatment of GBM located in the right frontal lobe. The radiotherapy plan map was obtained from the electronic medical records system (EMR) and was composed of representative **(a)** axial, **(b)** coronal, and sagittal (not presented) CT images (note; arrows represent the iso-dose line of 3600 cGy). In this patient, **(c)** the right thalamus (dashed circle) was designated as a Area_H_, whereas **(d)** the left frontal white matter (dashed circle) was considered a Area_L_ (presented images: pre-contrast T1WI).

For the subgroup analysis of the R1 value changes in other than peri-tumoral areas (i.e. GP, thalamus, and frontal, parietal and temporal white matter), the measured regions located within 50–100% iso-dose lines of the maximum radiation dose were defined as high radiation dose areas (Area_H_), whereas the areas outside Area_H_ were considered low radiation dose areas (Area_L_)(Fig 4). We also calculated the R1 ratio (R1 after radiation / R1 before radiation) in each area of the brain.

To evaluate the relationship between the R1 change and radiotherapy type, the 44 patients were divided into two groups according to radiotherapy type: routine vs. hypofractionated radiotherapy. In addition, study population was also divided into two groups according to the timing of post-radiation MRI: before the adjuvant temozolomide vs. after the completion of temozolomide.

To estimate long-term changes in the R1 value within the radiation field, subgroup analysis was performed. Among 44 patients, 10 patients had additional follow-up brain MR images that include the R1 map images and had no suspicious finding for tumor recurrence. In this group, the R1 ratio was calculated twice: once as the post-radiation R1 ratio (R1 after radiation / R1 before radiation) and once as the final R1 ratio (R1 of last MR imaging / R1 before radiation).

### Evaluation of renal and hepatic function

Serum chemistry tests results, which were performed within two weeks after post-radiation brain MR imaging and associated with renal and hepatic function, were also obtained. The estimated glomerular filtration rate (eGFR; mL/min/1.73m^2^) was measured using the Modification of Diet in Renal Disease (MDRD) equation. Abnormal hepatic function was defined by one or more abnormal serum concentrations of aspartate aminotransferase (>40 IU/L), alanine aminotransferase (>40 IU/L), gamma-glutamyl transpeptidase (>63 IU/L), or total bilirubin (>1.2 mg/dL).

### Statistical analysis

The Kolmogorov-Smirnov test was used to determine if the non-categorical variables of the clinical characteristics, the R1 value from each brain area, and the R1 ratio were distributed normally. Data with a normal distribution were compared between two groups using paired or unpaired Student’s t tests, and for data that did not follow a normal distribution, the Mann-Whitney U test or Wilcoxon test was applied.

Univariate linear regression analyses were performed to examine independent factors associated with the R1 value changes. Age, sex, Gd dose, time interval between two MR imaging sessions, radiotherapy type, hepatic function, and eGFR were designated as input variables. Multiple linear regression analysis (stepwise) using method of least squares was performed. Variables with a P-value less than 0.05 were entered, and variables with a P-value greater than 0.1 were removed from multiple regression analysis.

The intraclass correlation coefficient, Bland-Altman plotting, and coefficient of variation were used to assess inter-observer reproducibility. The chi-squared tests were performed to find the differences in frequency of hypofractionated radiotherapy according to variables.

All statistical analyses were conducted using MedCalc (Version 15.2, Ostend, Belgium). Statistical significance was defined as a P-value less than 0.05.

## Results

Table 1 summarizes basic information in 44 patients (mean age 54.3±15.2 years). The mean radiation dose was 57.0±7.2 Gy, and the mean administered Gd dose between two MR imaging sessions was 5.1±1.8 vials (range: 2–11; 1 vial = 7.5 mmol/7.5 mL of gadolinium). The results of intraclass correlation coefficient, coefficient of variation, and Bland-Altman plot were described in the S1 Table and S2 Fig.

**Table 1:** Basic information regarding patients, target lesions, treatment, and imaging work-up in 44 patients with supratentorial GBM.

**1. Age** (years; mean±SD)	54.3±15.2
**2. Sex** (number)	
Male	29
Female	15
**3. Location** (number)	
Right (n = 21)	Frontal: 8
	Parietal: 5
	Temporal: 3
	Thalamus: 1
	Multi-lobes: 4
Left (n = 21)	Frontal: 1
	Parietal: 2
	Temporal: 9
	Multi-lobes: 9
Bilateral (n = 2)	Frontal: 2
**4. Time interval between pre- and post-RT MR imaging session** (months; mean±SD)	4.2±2.1
**5. Radiation dose** (Gy; mean±SD)	
Total (n = 44)	57.0±7.2
RT_R_ (n = 33)	61.1±1.9
RT_H_ (n = 11)	45.0
**6. Timing of post-RT MR imaging** (number)	
before the adjuvant TMZ	38
after the completion of adjuvant TMZ	6
**7. Mean Gd dose** (vials; mean±SD)	5.1±1.8
**8. Patients using corticosteroid at the time of post-RT MR imaging** (number)	2
**9. eGFR** (mL/min/1.73 m^2^; mean±SD)	
Total (n = 44)	93.8±21.5
eGFR ≥90 (n = 22)	110.2±17.3
eGFR <90 (n = 22)	77.5±9.4
**10. Hepatic function** (number)	
Normal	34
Abnormal	10
**11. Patients who diagnosed as disease progression** (number)	
at 1 year after the GTR	18
at 2 years after the GTR	33

Note: GBM = glioblastoma multiforme, SD = standard deviation, RT = radiotherapy, MR = magnetic resonance, RT_R_ = routine radiotherapy, RT_H_ = hypofractionated radiotherapy, TMZ = temozolomide, Gd = gadolinium, eGFR = estimated glomerular filtration rate, Gy = Gray, GTR = gross total removal

### R1 value in each area of the brain

The R1 values in peri-tumoral areas were significantly increased after radiotherapy (0.7901±0.0977 [mean±SD] vs. 0.8146±0.1064, P <.01). There was no significant R1 value increase in areas other than the peri-tumoral region, including the bilateral thalami and GPs. Table 2 summarized the R1 value changes in each area after radiotherapy.

**Table 2:** R1 of each brain area and peri-tumoral area.

Measured Area	Pre-RT R1	Post-RT R1	P-value
	Mean±SD	Mean±SD	
Right thalamus	0.4882±0.0327	0.4819±0.0333	0.29
Left thalamus	0.5086±0.0332	0.5081±0.0350	0.92
Right GP	0.6235±0.0473	0.6248±0.0334	0.89
Left GP	0.6385±0.0441	0.6339±0.0420	0.70
Right frontal WM	0.8749±0.0882	0.8827±0.0720	0.55
Left frontal WM	0.8815±0.0922	0.8831±0.0744	0.91
Right parietal WM	0.7282±0.0547	0.7376±0.0461	0.35
Left parietal WM	0.7889±0.0425	0.8081±0.0616	0.09
Right temporal WM	0.7885±0.0500	0.7776±0.0653	0.24
Left temporal WM	0.8400±0.0784	0.8109±0.0633	0.01
Peri-tumoral area	0.7901±0.0977	0.8146±0.1064	<.01

Note: RT = radiotherapy, R1 = R1 value of tissue, SD = standard deviation, GP = globus pallidus, WM = white matter.

The R1 value of contralateral white matters in the Area_L_ did not change significantly (0.8222±0.0645 vs. 0.8121±0.0593, P = .24) before and after radiotherapy, and the R1 ratio was significantly higher in the peri-tumoral areas than in the contralateral white matter (1.0317±0.0698 vs. 0.9917±0.0647, P <.01).

### R1 value in the Area_H_ vs. Area_L_

The R1 ratio was significantly higher in the Area_H_ than in the Area_L_ (1.0055±0.0654 vs. 0.9882±0.0642; P <.01). Although there was no statistically significant R1 value increase in each brain area, the GP exhibited a tendency of increasing R1 according to radiation dose (1.0149±0.0719 vs. 0.9839±0.0676; P = .06; Table 3).

**Table 3:** R1 change in each brain area according to radiation dose.

Measured Area	Area_H_	Area_L_	P-value
	R1 ratio* (mean±SD)	R1 ratio (mean±SD)	
Frontal WM	1.0269±0.0909	1.0010±0.0991	0.23
Parietal WM	1.0366±0.0717	1.0089±0.0787	0.15
Temporal WM	0.9600±0.0533	0.9815±0.0807	0.47
Thalamus	1.0047±0.0618	0.9833±0.0819	0.18
GP	1.0149±0.0719	0.9839±0.0676	0.06
Overall area	1.0055±0.0654	0.9882±0.0642	<0.01

Note: Area_H_ = high radiation dose area, Area_L_ = low radiation dose area, SD = standard deviation, WM = white matter, GP = globus pallidus

* R1 ratio = R1 value after radiation / R1 value before radiation.

### R1 value according to radiotherapy type and temozolomide dose

The R1 ratio in the peri-tumoral areas was lower in the 11 patients who underwent hypofractionated radiotherapy than in the 33 patients who underwent routine one (0.9913±0.0740 vs. 1.0463±0.0633, P = .08), although this difference was not statistically significant.

In univariate analyses, radiotherapy type (P = .02) was found to be a single significant factor related to the R1 value increase in the peri-tumoral areas. Other variables were not related with peri-tumoral R1 value increase (Table 4). Subsequent multiple linear regression analysis revealed that routine radiotherapy (coefficient of determination R^2^ = 0.1202, P = .02) was the only significant factor for R1 value increase in the peri-tumoral areas.

**Table 4:** Univariate linear regression analyses regarding the peri-tumoral R1 changes.

Variables	Regression Coefficient	Standard Error	95% C.I	P-value
**Age**	-0.001132	0.0006816	-0.002508 to 0.0002433	0.10
**Sex**	0.004652	0.02237	-0.04048 to 0.04979	0.84
**Gd dose**	-0.0002328	0.006051	-0.01244 to 0.01198	0.97
**Time interval**	0.001584	0.005144	-0.008796 to 0.01196	0.76
**RT type**	0.1101	0.04595	0.01732 to 0.2028	0.02
**Hepatic function**	0.003787	0.02530	-0.04728 to 0.05485	0.88
**eGFR**	0.0005403	0.0004914	-0.0004514 to 0.001532	0.28

Note: C.I = confidence interval, Gd = gadolinium, RT = radiotherapy, eGFR = estimated glomerular filtration rate.

S2 Table summarizes the results of the chi-squared tests regarding the frequency of hypofractionated radiotherapy according to the variables.

In addition, mean R1 ratio was not significantly different between two groups with different doses of temozolomide (1.0334±0.0742 vs. 1.0274±0.0284; P = 0.72).

### Long-term changes in R1 value (in 10 patients)

The mean administered Gd dose and the time interval between the first and last brain MR imaging sessions were 9.1±0.9 vials (range: 8–11) and 11.2±2.6 months (range: 7–15.5), respectively. The mean peri-tumoral R1 at pre- and post-radiation and last MR imaging were 0.7768±0.1089, 0.7800±0.1141, and 0.8061±0.1194, respectively. The mean post-radiation R1 ratio was 1.0073±0.1040, and the mean final R1 ratio was 1.0384±0.0909. The mean final R1 ratio was not significantly different from either the mean post-radiation R1 ratio (P = .31) or mean R1 ratio of the 44 patients (P = .91).

## Discussion

Radiation induced BBB disruption [9–15] is well established radiation related changes in the brain, and this could result in accelerated passage of materials through the BBB. We hypothesized Gd could be one of those substances, and this phenomenon could be evaluated quantitatively by measuring R1 value of brain parenchyma before and after radiotherapy.

In the present study, significant R1 value increase (i.e. T1 shortening) in the brain parenchyma in the Area_H_ after several rounds of injection of macrocyclic GBCA was observed. However, no significant change in the R1 value was noted in the Area_L_ of the brain, even after similar exposure to the macrocyclic GBCA. Another important finding is that the radiotherapy type was a significant factor for T1 shortening in the peri-tumoral area of the brain after multiple injections of the macrocyclic GBCA.

A variety of conditions can induce T1 hyperintensity in the brain, including history of irradiation [21], multiple sclerosis [22], paramagnetic substances in patients with intracranial hemorrhage or Wilson’s disease, and calcification [23–25]. Among them, we concluded that radiation-induced BBB weakness [10] and subsequent Gd accumulation within the radiation field was the cause of T1 shortening.

Even if radiation-induced superoxide dismutase [22] or vascular damage and subsequent ischemic change of the brain tissue [26] can also induce T1 hyperintensity of the brain, these changes would not persistent for longer than a year according to the quantitative study by Fujioka et al [27]. T1 hyperintensity tended to decrease gradually several months after the ischemic event in their study [27].

According to qualitative studies performed by Steen et al [14, 15], radiation-induced T1 shortening of white matter in pediatrics became significant 6 months after radiotherapy and had persisted through a year, and this change was obvious in the area exposed to radiation more than 20 Gy. Their results [14, 15] also revealed that there was neither significant T1 shortening in brain tissue within 16 weeks after radiation nor T1 shortening difference between cerebral white matter exposed to 40–50 Gy and more than 54 Gy.

In our study, by contrast, the T1 shortening of peri-tumoral region was observed shorter than 16 weeks after the radiotherapy, and degree of T1 shortening was quite different between routine radiotherapy and hypofractionated groups. Considering our results of early, radiation dose-dependent T1 shortening, observed R1 value increase within the radiation field might truly reflect Gd deposition and retention in that area rather than accumulation of radiation induced substances. In addition, because radiotherapy generally induces demyelination and subsequent T1 prolongation [28–30], the substantial amount of Gd might deposit within the radiation field to overcome the T1 prolongation induced by radiation.

Although it was not statistically significant, the GP also showed T1 shortening with increasing radiation dose (Area_H_ vs. Area_L_), even though we administered macrocyclic GBCA. These results further suggest that the relatively low integrity and increased vulnerability of the BBB at the GP were responsible for the characteristic T1 shortening in this area in patients administered GBCAs.

Notably, younger patients had a tendency of T1 shortening within the radiation field on univariate linear regression analysis (P = .10). We thought that this finding is also resulted from the difference in the frequency of radiotherapy type between the younger and older patient groups (P = .02). This result also supports a dose-dependent relationship between radiation and the T1 shortening.Interestingly, multiple regression analysis revealed that the administered gadolinium dose was not associated with T1 shortening. One possible explanation of this finding is that damage to the BBB could allow circulating gadolinium to pass through the weakened BBB; after repair of the BBB, the gadolinium could not pass through the BBB and was retained in that area.

Our experience about the impact of Gd on neuronal tissues has been limited, and there has been no definite evidence that accumulated Gd can affect brain function [31]. Although radiologists should not fall in a kind of “Gd-phobia”, several studies [32–34] implied we could not totally exclude the possibility of neurotoxicity of Gd. If further studies reveal that accumulation of Gd in the brain parenchyma has an effect on neuronal function, cautious Gd administration or radiation dosage adjustment in patients for whom intracranial radiotherapy is planned could be suggested based on our results.

Our study has several strengths. First, we used the absolute R1 value rather than the signal intensity ratio. Although the inversion recovery (IR) method is considered the gold standard for T1 mapping [35], we obtained evidence about T1 signal intensity changes by presenting quantitative values using variable flip angle methods. Second, we identified a homogeneous study population diagnosed with GBM who underwent standardized treatment. In addition, the entire study population underwent brain MR imaging using gadobutrol, and the R1 maps were acquired using a single MRI machine (Verio), except for one patient, thus reducing random effects in our study.

Our study also has several limitations. First, there is the inevitable limitation that this was a retrospective study. Second, we selected GBM patients as our study population. Although that selection provided a homogeneous study group in terms of disease and treatment, long-term follow-up brain MR imaging was limited because the reported median survival of glioblastoma patients treated with radiotherapy plus temozolomide range from 9.7 to 14.6 months [36–38]. Thus, there was limited evaluation of long-term R1 changes in our patients group. Third, we only analyzed brain MR images and did not sample the neuronal tissue where T1 shortening was observed. Further histological studies including mass spectrometry or electron microscopy might be needed to clarify the gadolinium deposition within the radiation field. Fourth, there might be association of T1 shortening and temozolomide chemotherapy. However, there was no significant difference between R1 ratios of post-radiation MR imaging before and after the completion of adjuvant temozolomide. In addition, if temozolomide affects T1 shortening in the brain, global brain T1 shortening is likely to occur. However, our results show that T1 shortening is predominantly observed inly in peri-tumoral areas, which were located within 50–100% iso-dose lines of maximum radiation dose.

In conclusion, our study demonstrated T1 shortening within the radiation field, which might suggest that radiotherapy can enhance Gd accumulation in the brain parenchyma by damaging the BBB. Gd accumulation within the DN and GP might be related to the integrity of the BBB in those areas, and further studies are warranted to investigate the relationship between the BBB and Gd deposition.

## Supporting information

S1 Table Inter-observer reproducibility of the R1 measurements. (DOCX)

S2 Table The frequency of RT_H_ according to variables. (DOCX)

S1 Fig Measurement and comparison of the R1 value in the brain. (DOCX)

S2 Fig Bland-Altman plots showing inter-observer reproducibility between the two observers. (DOCX)

S1 File Basic information and R1 value of 44 GBM patients at Seoul National University Hospital. (XLSX)
