# Supplementary material for: Does radiation therapy increase gadolinium accumulation in the brain?: Quantitative analysis of T1 shortening using R1 relaxometry in glioblastoma multiforme patients
Source: PLoS One. 2018 Feb 14;13(2):e0192838. doi: 10.1371/journal.pone.0192838 (PMC5812640; doi:10.1371/journal.pone.0192838)
Supplement: S1 Table — (DOCX) [file pone.0192838.s001.docx]

**Supporting Information**

**S1 Table.** Inter-observer reproducibility of the R1 measurements

| **Measured Area** | **ICC (95% CI)^*^** | **CV** |
| --- | --- | --- |
| Right thalamus | 0.8945 (0.8047 to 0.9431) | 3.5790 |
| Left thalamus | 0.9157 (0.8439 to 0.9546) | 2.6710 |
| Right GP | 0.9720 (0.9464 to 0.9854) | 1.8623 |
| Left GP | 0.9552 (0.9135 to 0.9769) | 1.8809 |
| Right frontal WM | 0.9669 (0.9382 to 0.9823) | 2.3669 |
| Left frontal WM | 0.9809 (0.9641 to 0.9899) | 1.9335 |
| Right parietal WM | 0.9815 (0.9628 to 0.9908) | 1.4467 |
| Left parietal WM | 0.9711 (0.9406 to 0.9860) | 1.8102 |
| Right temporal WM | 0.8880 (0.7838 to 0.9422) | 3.8219 |
| Left temporal WM | 0.8510 (0.7068 to 0.9245) | 4.2971 |
| Peri-tumoral area | 0.9532 (0.9146 to 0.9744) | 2.0605 |
| Overall areas | 0.9437 (0.9318 to 0.9535) | 2.6570 |

Note: ICC=intraclass correlation coefficient, CV=coefficient of variation, CI=confidence interval, GP=globus pallidus, WM=white matter.

* ICC values were categorized as follows: <0.40, poor; 0.40-0.59. fair; 0.60-0.74, good; and >0.74, excellent.
