# Supplementary material for: Does radiation therapy increase gadolinium accumulation in the brain?: Quantitative analysis of T1 shortening using R1 relaxometry in glioblastoma multiforme patients
Source: PLoS One. 2018 Feb 14;13(2):e0192838. doi: 10.1371/journal.pone.0192838 (PMC5812640; doi:10.1371/journal.pone.0192838)
Supplement: S2 Table — (DOCX) [file pone.0192838.s002.docx]

**Supporting Information**

**S2 Table.** The frequency of RT_H_ according to variables

| **Variables** |  | **Number of patients** | **Number of RT_H_** | **P-value*** |
| --- | --- | --- | --- | --- |
| **Age** | Young  Old | 19  25 | 1  10 | 0.02 |
| **Sex** | Male  Female | 29  15 | 8  3 | 0.85 |
| **Gd dose** | High  Low | 13  31 | 3  8 | 0.85 |
| **Time interval** | Long  Short | 7  37 | 3  8 | 0.48 |
| **Hepatic function** | Normal  Abnormal | 34  10 | 9  2 | 1.000 |
| **eGFR** | High  Low | 18  26 | 3  8 | 0.48 |

Note: RT_H_=hypofractionated radiotherapy, Gd=gadolinium, eGFR=estimated glomerular filtration rate.

Note: The thresholds for continuous variables (age, gadolinium dose, time interval, and eGFR) were defined as the arithmetic mean of those variables.

* P-value of chi-squared tests.
