# Supplementary material for: Does radiation therapy increase gadolinium accumulation in the brain?: Quantitative analysis of T1 shortening using R1 relaxometry in glioblastoma multiforme patients
Source: PLoS One. 2018 Feb 14;13(2):e0192838. doi: 10.1371/journal.pone.0192838 (PMC5812640; doi:10.1371/journal.pone.0192838)
Supplement: S1 Fig — (DOCX) [file pone.0192838.s003.docx]

**Supporting Information**

**S1 Fig.** Measurement and comparison of the R1 value in the brain

For the R1 measurements, ROIs were drawn at peri-tumoral areas and the bilateral frontal, parietal, and temporal white matters. ROIs were also drawn at the bilateral GPs and thalami. Analyses of this study were performed in three categories; 1) comparison of the R1 value before and after radiation, 2) comparison of the R1 ratio (R1 after radiation / R1 before radiation) in each patient and each brain area, and 3) comparison according to radiotherapy type.
