# Supplementary material for: Does radiation therapy increase gadolinium accumulation in the brain?: Quantitative analysis of T1 shortening using R1 relaxometry in glioblastoma multiforme patients
Source: PLoS One. 2018 Feb 14;13(2):e0192838. doi: 10.1371/journal.pone.0192838 (PMC5812640; doi:10.1371/journal.pone.0192838)
Supplement: S2 Fig — (DOCX) [file pone.0192838.s004.docx]

**Supporting Information**

**S2 Fig.** Bland-Altman plots showing inter-observer reproducibility between the two observers.
